# A Case on Papillary Thyroid Carcinoma With Intraluminal Tracheal Extension: A Malaysian Experience and Literature Review

**DOI:** 10.7759/cureus.61712

**Published:** 2024-06-05

**Authors:** Alex Zxi Jian Ho, Azwan Halim Abdul Wahab, Atikah Rozhan

**Affiliations:** 1 Department of Otolaryngology, Head and Neck Surgery, International Islamic University Malaysia, Kuantan, MYS

**Keywords:** tracheal resection and anastomosis, tracheal resection, extrathyroidal extension, tracheal invasion, papillary carcinoma of thyroid

## Abstract

Papillary thyroid carcinoma (PTC) is a common malignancy originating from the thyroid gland. In rare cases, it can invade the trachea, resulting in airway obstruction. Subsequent surgical planning may be complicated as the technique selected depends on a case-by-case basis. Here, we report a case of PTC with tracheal involvement and a literature review on the latest surgical options.

A 56-year-old gentleman presented with an anterior neck swelling of 3 x 3 cm for 3 months. Flexible endoscopy showed irregular mass in the subglottic region. Subsequent aspiration for cytology confirmed a diagnosis of PTC. Neck contrast enhanced computed tomography showed an ill-defined lesion in the right thyroid (3.1 x 3.8 x 2.9 cm) with a subtle irregularity of the adjacent tracheal wall suggestive of infiltration. The findings indicated a clinical staging of cT4aN0M0 (Stage III) with Shin’s staging of Stage IV. The patient underwent a total thyroidectomy and a single-stage partial cricoid-tracheal resection with anastomosis. There were no immediate post-operative complications reported. Unfortunately, the patient suffered from pulmonary embolism, which eventually resulted in his demise. A subsequent histopathology report confirmed the diagnosis of PTC.

Surgical planning for such cases may be complicated. The risk of recurrent laryngeal nerve injury is increased as the site of resection is close to the nerve. Multiple intraoperative nerve monitoring systems may be required. Meticulous planning of intraoperative airway management is needed as a large intraluminal tumor may interfere with intubation. Generally, extensive tracheal invasion would require radical surgical approaches such as circumferential resection and total laryngectomy. Less extensive cases can be treated with shave excision or window resection.

PTC with tracheal invasion is an uncommon condition, and surgical excision is indicated for cases with high Shin’s staging.

## Introduction

Papillary thyroid carcinoma (PTC) is a common malignancy of the thyroid gland with a common metastasis to surrounding cervical lymph nodes. Tracheal invasion is a rare complication that has the potential to cause airway obstruction. An incidence of 1% to 13% has been reported for tracheal involvement in such malignancies [1,2]. The presence of tracheal involvement complicates the subsequent surgical management, leading to increased morbidity and mortality. Here, we report a case of PTC with tracheal involvement, detailing our experience as well as a literature review on the latest surgical management options. This article was previously presented as a meeting abstract at the International Virtual Medical Research Symposium 2023 on December 7, 2023.

## Case presentation

**Figure 1:**
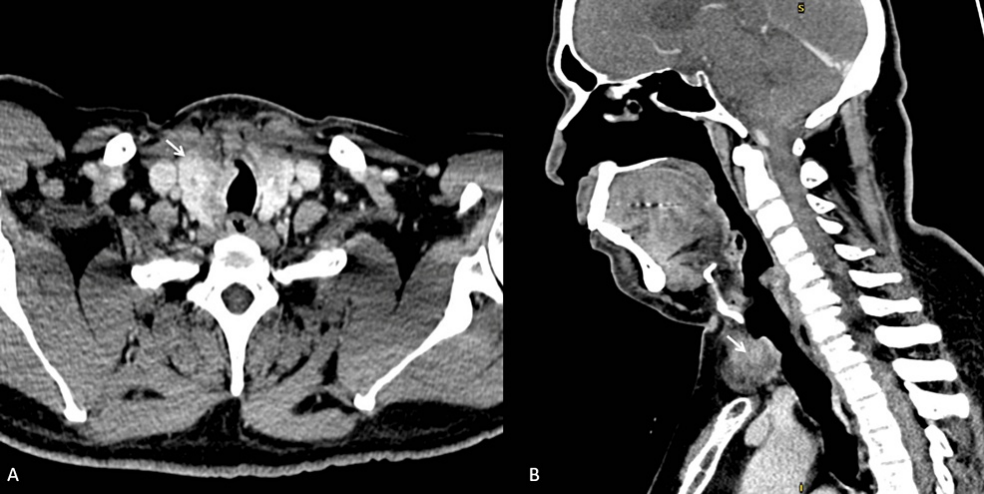
Computed tomography of the neck showing thyroid lesion with tracheal involvement A: Axial cut with arrow showing tumor with tracheal cartilage invasion B: Sagittal cut with arrow showing tumor

A 56-year-old gentleman presented with an anterior neck swelling of 3 x 3 cm. This was associated with increased effort tolerance and a feeling of discomfort upon lying flat. Flexible endoscopy showed irregular mass in the subglottic region. A subsequent aspiration for cytology revealed a diagnosis of PTC. Contrast enhanced computed tomography (CECT) of the neck showed a 3.1 x 3.8 x 2.9 cm ill-defined lesion in the right thyroid lobe abutting the trachea, with mass effect displacing it to the left (Figure 1).

A subtle irregularity of the tracheal wall was suggestive of tumor infiltration. Otherwise, multiple sub-centimeter cervical lymph nodes were seen at bilateral levels II, III, and IV regions.

The findings indicated a clinical staging of cT4aN0M0 (stage III) with Shin’s staging of stage IV. Subsequently, the patient underwent a total thyroidectomy with a single-stage partial cricoid-tracheal resection and anastomosis. He required intensive care post-surgery and was planned for intubation for 5 days to allow tracheal wound healing. Intravenous corticosteroids were given to prevent intraluminal granulation tissue formation. There were no immediate postoperative complications reported. However, the patient sustained pulmonary embolism, likely due to prolonged surgery and immobility, which eventually resulted in his demise. A subsequent histopathology report confirmed the diagnosis of PTC.

## Discussion

**Figure 2:**
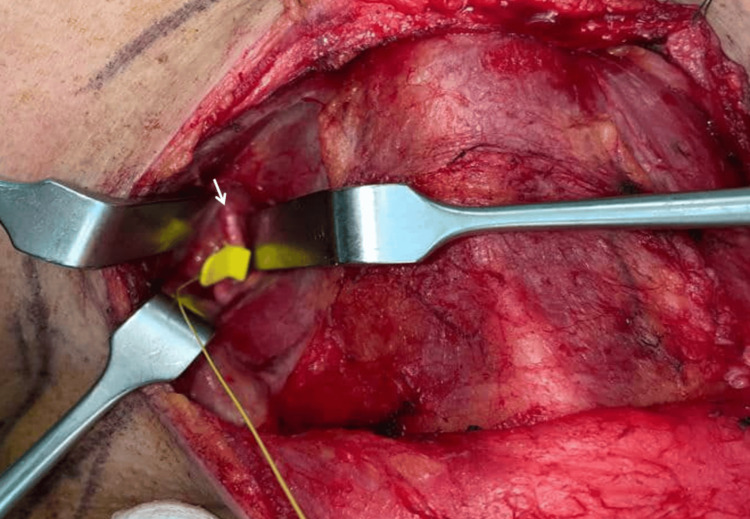
Localization of the right vagus nerve with probe attached Arrow: Right vagus nerve

We performed a total thyroidectomy with single-stage partial cricoid-tracheal resection and anastomosis for a case of PTC with tracheal invasion (Shin's stage 4). Upon neck incision, the tracheal cartilage was identified for the placement of a probe as part of Taiwan’s method of nerve monitoring. The left vagus nerve was identified in the region bordered by the superior omohyoid, sternocleidomastoid muscles, and midline of the neck. A probe was attached for continuous nerve monitoring. Next, the left recurrent laryngeal nerve (RLN) was identified and preserved, followed by a left thyroidectomy. The procedure was repeated on the right, with the vagus and right RLN identified and preserved (Figure 2). The right thyroid mass was noted to be embedded into the laryngeal framework in the right cricoid-tracheal region. It was then cut through, leaving tumor remnants.

**Figure 3:**
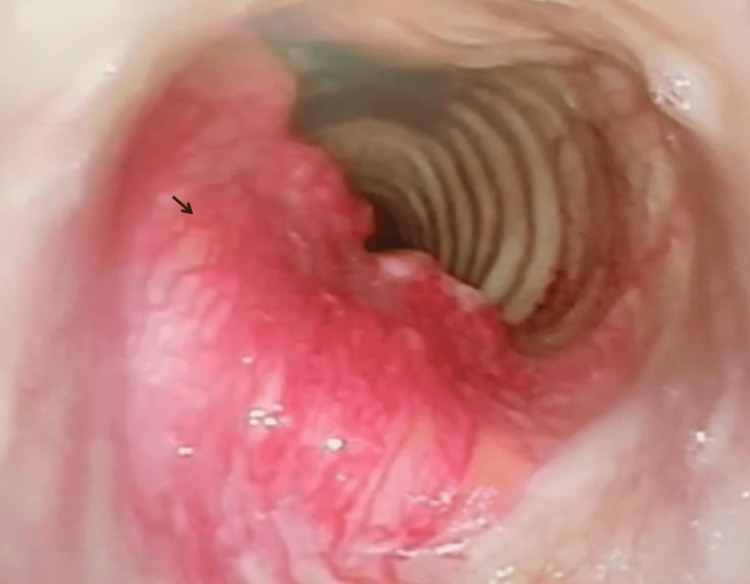
Endoscopic view of tumor with trachea involvement Arrow: Tumor

**Figure 4:**
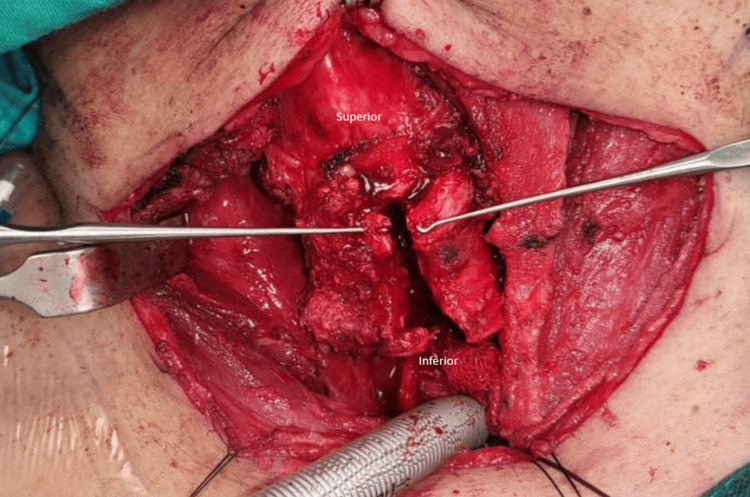
Midline cricoid split with inferior trachea extension

**Figure 5:**
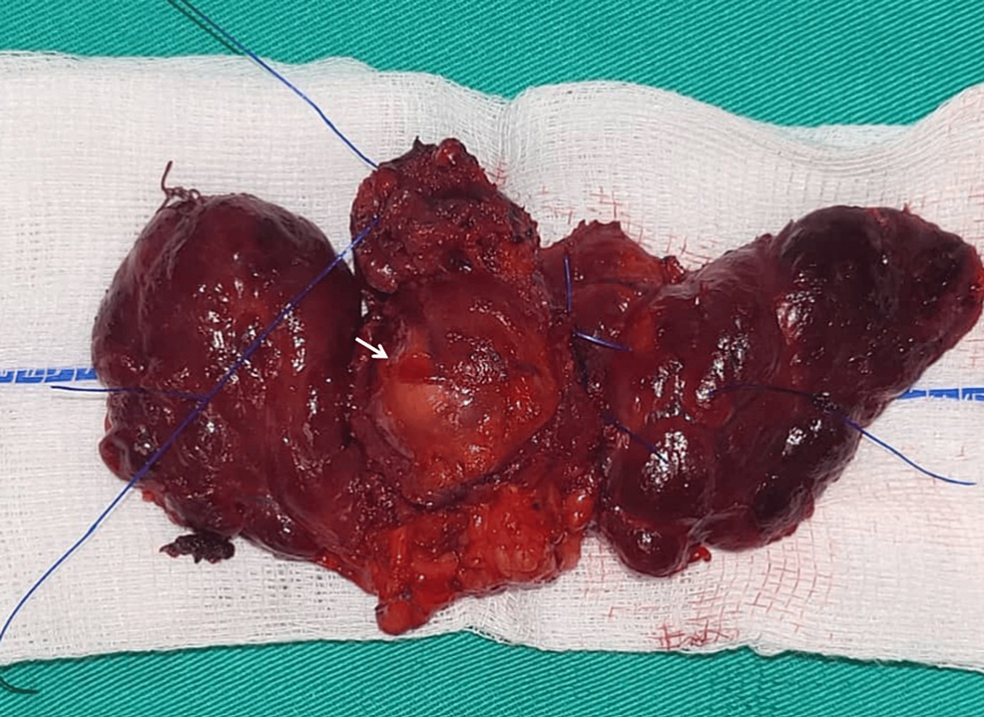
Gross thyroid, resected cricoid, and trachea specimen Arrow: Intraluminal tumor

Following that, the patient was put under intermittent apnea as we performed a direct laryngoscopy and tracheoscopy to assess intraluminal tumor involvement. The intraluminal tumor length measured 1.9 cm, approximately 1.8 cm inferior to the vocal cords (Figure 3). Markings were made on the outer trachea using a needle as guidance. Reintubation was done with an endotracheal tube cuff positioned distal to the tumor level. The cricoid was split in the midline and extended inferiorly until complete visualization of the tumor (Figure 4). The anterior half of the cricoid with the involved tracheal segment measuring approximately 4 cm was removed (Figure 5). Stay sutures were placed at the lateral borders of the trachea to prevent inferior displacement. Both ends were anastomosed with interrupted sutures. (Posterior: 3 sutures; right/left) Anterolateral: 2 sutures each. Before closure, the Valsalva maneuver was performed with no evidence of leakage demonstrated.

Generally, extrathyroidal extension in PTC indicates a poor prognosis. This is particularly so in cases of aerodigestive tract invasion [3]. Cases as such were described as having increased biological tumor aggressiveness secondary to reduced tumor suppressor adhesion molecule expression (e.g., E-cadherin) [4]. The TNM classification categorized extrathyroidal invasion of PTC with aerodigestive tract invasion as stage T4a [5].

Tracheal invasion was initially hypothesized to be an extension of a metastatic tumor from peritracheal lymph nodes. However, a newer study postulated that tracheal invasion was due to direct invasion of the primary tumor. This may occur through a potential weakness in the tracheal wall at sites of vessel penetration [6]. Shin et al. further described a classification of direct tumor invasion based on the depth of tracheal involvement [6].

In cases of superficial invasion with no cricoid or tracheal lumen breach, tracheal-preserving procedures such as shave excision were deemed appropriate [7]. Shave excision removes a superficially invading tumor with the preservation of normal cricoid and tracheal tissues [8]. Upon comparing shave excision to radical resection in cases of minimal invasion, similar survival rates were demonstrated [8].

However, complete resection is recommended when intraluminal involvement is present [4]. The approach to surgical intervention depends on the depth and extent of tumor involvement. In unilateral laryngotracheal invasion, window resection is recommended for cases with less than 50% tracheal ring circumference involvement spanning less than four tracheal rings [9]. Reconstruction is indicated when tumor involvement is more than one-third tracheal circumference with more than 2.5 cm in length [7]. Previously described techniques include the use of the sternocleidomastoid, pectoralis major muscle, and anterior cervical flap [9]. Vertical hemi-laryngectomy with repair is an alternative surgical consideration for extensive unilateral invasion [7].

Advanced tracheal invasion, such as in Shin’s Stage 4 cases, will require circumferential resection. Various studies suggest that cases with tumor infiltration of more than 50% tracheal ring circumference up to six rings would benefit from circumferential resection with anastomosis [7,9]. In cases with posterior trachea involvement, a total laryngectomy or laryngo-pharyngectomy may be required [7].

Our case had tumor infiltration involving more than 50% of the tracheal and cricoid circumference. The length involved was approximately 2 tracheal rings (1.9 cm). Therefore, it is a suitable case for partial crico-tracheal resection with anastomosis to achieve complete tumor removal. Resection in the cricothyroid region increases the risk of RLN injury as the nerve enters the larynx at this level. This could affect the outcome of surgery, as the risk of nerve injury has to be balanced with the risk of inadequate resection. In our case, cricoid resection was done meticulously with two intraoperative nerve monitoring systems. There was no intraoperative loss of signal, and the resection margins were clear. Incomplete tumor resection may lead to higher recurrence and worse survival rates [10].

Another issue to consider is intraoperative airway management, as a large intraluminal tumor may interfere with intubation. In such cases, excision of the intraluminal tumor may be performed via endoscopic technique. Methods such as endoscopic laser resection have been described in previous literature [11]. In cases where complete tracheal excision is not attainable, adjuvant radioiodine therapy should be given. Karkos et al. described a successful case of total thyroidectomy and endoscopic laser resection of a tumor with preservation of tracheal cartilages for a case of PTC. No recurrence was noted up to nine months of follow-up [11].

Unfortunately, we were unable to evaluate the long-term outcome of our patient due to unfortunate complications. Nevertheless, surgical intervention in such cases is a viable option to ensure complete disease removal. Future research into newer techniques and approaches would be beneficial, especially in cases with complicated tracheal involvement.

## Conclusions

Surgical intervention in the management of PTC with tracheal involvement is a viable option to ensure complete disease removal. The choice of surgical technique or approach should be tailored specifically to each patient. In the future, further development of different techniques and approaches would be beneficial, especially in cases with complicated tracheal involvement.

## References

[1] Choi MJ, Kang H (2021). CT findings of central airway lesions causing airway stenosis-visualization and quantification: a pictorial essay. Taehan Yongsang Uihakhoe Chi.

[2] Matsumoto F, Ikeda K (2021). Surgical management of tracheal invasion by well-differentiated thyroid cancer. Cancers (Basel).

[3] Ito Y, Tomoda C, Uruno T (2006). Prognostic significance of extrathyroid extension of papillary thyroid carcinoma: massive but not minimal extension affects the relapse-free survival. World J Surg.

[4] McCaffrey JC (2006). Aerodigestive tract invasion by well-differentiated thyroid carcinoma: diagnosis, management, prognosis, and biology. Laryngoscope.

[5] Paleri V, Mehanna H, Wight RG (2010). TNM classification of malignant tumours 7th edition: what's new for head and neck?. Clin Otolaryngol.

[6] Shin DH, Mark EJ, Suen HC (1993). Pathologic staging of papillary carcinoma of the thyroid with airway invasion based on the anatomic manner of extension to the trachea: a clinicopathologic study based on 22 patients who underwent thyroidectomy and airway resection. Hum Pathol.

[7] Kebebew E, Clark OH (2003). Locally advanced differentiated thyroid cancer. Surg Oncol.

[8] Moritani S (2015). Surgical management of cricotracheal invasion by papillary thyroid carcinoma. Ann Surg Oncol.

[9] Zhang J, Fu C, Cui K, Ma X (2019). Papillary thyroid carcinoma with tracheal invasion: A case report. Medicine (Baltimore).

[10] Parida PK, Herkal K, Preetam C, Pradhan P, Samal DK, Sarkar S (2022). Analysis of pattern of laryngotracheal invasion by papillary thyroid carcinoma and their management: our experience. Indian J Otolaryngol Head Neck Surg.

[11] Karkos PD, Koskinas IS, Tsiropoulos G, Goupou E, Hatzibougias D (2021). Metastatic tracheal tumor of thyroid origin: endoscopic diode laser treatment combined with thyroidectomy, radioactive iodine, and radiotherapy. Ear Nose Throat J.

